# Transportation Barrier in Rural Older Adults’ Use of Pain Management and Palliative Care: Systematic Review

**DOI:** 10.1093/geroni/igaa057.1399

**Published:** 2020-12-16

**Authors:** Zainab Suntai, Chorong Won, Hyunjin Noh

**Affiliations:** University of Alabama, Tuscaloosa, Alabama, United States

## Abstract

Pain and symptom management is critical in ensuring quality of life for chronically or seriously ill older adults. However, while pain management and palliative care have steadily expanded in recent years, many underserved populations, such as rural older adults, experience barriers in accessing such specialty services, particularly due to transportation issues. The purpose of this systematic review is to examine the specific types of transportation-related barriers experienced by rural older adults in accessing pain and palliative care. Studies were searched through the following 10 databases: Abstracts in Social Gerontology, Academic Search Premier, CINAHL, MEDLINE, PsycINFO, SocINDEX with Full Text, Cochrane Database of Systematic Reviews, Nursing & Allied Health Database, Sociological Abstracts, and PubMED. Studies were chosen for initial review if they were written in English, full-text, included older adults in sample, and examined pain/palliative care/hospice, rural areas, and transportation. A total of 174 abstracts were initially screened, 15 articles received full-text reviews and eight met the inclusion criteria. Findings of the eight studies identified transportation-related issues as major access barrier to pain and palliative care among rural older adults: specifically, lack of public transportation; lack of special needs/wheelchair accessible vehicles; lack of reliable drivers; high cost of transportation services; poor road conditions; and remoteness to the closest pain and palliative care service providers. Results suggest that rural older adults have unique transportation needs due to the urban-centric location of pain and palliative care services. Implications for practice, policy and research with older adults are discussed.

